# Stream Mesocosm Experiments Show no Protective Effects of Calcium on Copper Toxicity to Macroinvertebrates

**DOI:** 10.1002/etc.5308

**Published:** 2022-03-21

**Authors:** Yuichi Iwasaki, Pete Cadmus, James Ranville, William H. Clements

**Affiliations:** ^1^ Department of Fish, Wildlife, and Conservation Biology Colorado State University Colorado Fort Collins USA; ^2^ Research Institute of Science for Safety and Sustainability National Institute of Advanced Industrial Science and Technology Ibaraki Tsukuba Japan; ^3^ Colorado Parks and Wildlife Colorado Fort Collins USA; ^4^ Department of Chemistry and Geochemistry Colorado School of Mines Colorado Golden USA

**Keywords:** Benthic macroinvertebrates, Trace metals, Freshwater toxicology, Water quality criteria, Biotic ligand model, Aquatic insects

## Abstract

Although the concept and modeling of metal bioavailability and toxicity have been well developed based largely on laboratory experiments with standard test species, additional evidence is required to demonstrate their applicability for macroinvertebrates typically found in natural lotic ecosystems. We conducted 10‐day stream mesocosm experiments to test the hypothesis that increased water hardness (in the present study, the calcium [Ca] concentration was increased by adding CaCl_2_) would mitigate the effects of copper (Cu) on natural benthic macroinvertebrate communities. Exposure of macroinvertebrate communities to 25 μg/L Cu for 10 days in stream mesocosm experiments resulted in significant decreases in total abundance, in number of taxa, and in abundance of many macroinvertebrate taxa. However, the addition of Ca to stream mesocosms and the associated increase in water hardness up to 250 mg/L CaCO_3_ did not mitigate these effects of Cu on macroinvertebrate communities. The results showed that the hardness‐based water quality criteria for Cu of the US Environmental Protection Agency were not protective under the conditions of relatively high hardness, low alkalinity, and circumneutral pH. In contrast, the water quality criteria based on the biotic ligand model predicted little protective effects of Ca on Cu toxicity, which is consistent with our results. Additional experiments are required to understand the influence of modifying factors on the toxicity of metals to macroinvertebrate communities. *Environ Toxicol Chem* 2022;41:1304–1310. © 2022 The Authors. *Environmental Toxicology and Chemistry* published by Wiley Periodicals LLC on behalf of SETAC.

## Graphical abstract

We examined whether increased water hardness (in the present study, calcium concentration) would mitigate the impacts of copper on natural benthic macroinvertebrate communities.

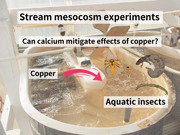

## INTRODUCTION

Managing the risks associated with the release of trace metals to freshwater ecosystems is a long‐standing global concern (Clements et al., 2021; Iwasaki & Ormerod, 2012; Luoma & Rainbow, 2008). Much effort has focused on the evaluation of how to incorporate metal bioavailability for achieving more science‐based risk assessment and management (Adams et al., 2020; Farley et al., 2015; Luoma & Rainbow, 2008; Meyer, 2002). Metal bioavailability and toxicity depend on water quality characteristics such as temperature, pH, water hardness, and dissolved organic matter (Allen et al., 1980; Meyer et al., 1999; Niyogi & Wood, 2004; Pinheiro et al., 2021). Ecological risk assessments of some metals account for factors that determine metal bioavailability (e.g., copper [Cu]: European Copper Institute, 2008 and nickel: European Chemicals Bureau, 2008), and the US Environmental Protection Agency (USEPA) has revised water quality criteria (WQC) for Cu (USEPA, 2007) based on the biotic ligand model (BLM: Mebane et al., 2020a; Paquin et al., 2002). However, BLMs need data not routinely collected during many water quality monitoring programs and also require complex speciation and modeling software. More recently, the USEPA released WQC for aluminum based on multiple linear regression models that account for metal bioavailability (Adams et al., 2020; Brix et al., 2020). Although BLM‐based WQC for Cu are available and the simpler multiple linear regression models perform well with Cu (Brix et al., 2017, 2021), at present, the vast majority of US states still rely on the hardness‐adjusted WQC. This disconnect between recommended policy and actual policy led us to develop the present study, to measure the effects of water hardness on Cu toxicity to a natural assemblage of stream benthic communities.

Benthic macroinvertebrates are frequently monitored and surveyed to assess the health of aquatic communities exposed to chemicals in natural environments (Namba et al., 2020; Rosenberg et al., 2008). However, although the importance of metal bioavailability has been well documented for aquatic species commonly used in laboratory toxicity tests (standard test species such as *Daphnia magna*), there is limited empirical evidence supporting this concept for common lotic macroinvertebrates that dominate stream ecosystems (e.g., mayflies, stoneflies, and caddisflies). Schmidt et al. (2010) and Iwasaki et al. (2013) showed the importance of considering metal bioavailability in predicting responses of macroinvertebrates in field surveys and mesocosm experiments. Several bioavailability‐adjusted models have been developed to estimate effects of metals on macroinvertebrate communities (Balistrieri et al., 2015; De Jonge et al., 2013; Fornaroli et al., 2018; Mebane et al., 2017; Stockdale et al., 2010); however, these studies did not directly examine the superiority of bioavailability‐adjusted models by comparing them with simpler approaches that did not consider bioavailability. Because the concept of bioavailability of trace metals such as Cu has been developed based largely on laboratory experiments with standard test species, obtaining additional experimental evidence is useful to demonstrate the applicability of this concept for macroinvertebrates typically found in natural lotic ecosystems.

We conducted stream mesocosm experiments to test the hypothesis that increased water hardness (in the present study, calcium [Ca] concentration) would mitigate the effects of Cu on natural benthic macroinvertebrate communities. Based on results from previous experiments (Clements et al., 2013), we selected a target concentration of Cu (25 μg/L) that would elicit significant effects on macroinvertebrate communities, and used multiple treatment levels of water hardness up to 250 mg/L CaCO_3_ by increasing Ca concentrations. The USEPA hardness‐adjusted environmental WQC for Cu were 3.2 and 19.6 μg/L at the water hardness values of 30 (controls) and 250 mg/L CaCO_3_, respectively (USEPA, 2002). We predicted that increased levels of Ca^2+^ would provide concentration‐dependent protective effects on benthic macroinvertebrates in the stream mesocosm experiments.

## MATERIALS AND METHODS

### Mesocosm experiments

We performed two stream mesocosm experiments using natural benthic communities collected from an unpolluted reference site (39°48′45.8″N, 105°29′53.6″W) on the North Fork of Clear Creek, a third‐order tributary to the Clear Creek watershed located near Blackhawk, Colorado (USA; see Cadmus et al., 2016 for a detailed site description). Following a previously described procedure (Clements, 1999; Clements et al., 2013, 2019; Cadmus et al., 2016), the mesocosm experiments were conducted in early fall, 2012. Plastic trays (10 × 10 × 6 cm) filled with pebble and cobble substrate collected onsite were placed in a riffle area for invertebrate colonization. Previous studies have shown that invertebrate communities colonizing these trays were similar to natural communities (Clements, 1999). Trays were collected on September 22, 2012 after 30 days of colonization and transported to the stream mesocosm facility located at the Colorado State University Stream Research Laboratory (Fort Collins, CO, USA). The facility consists of 18 stream mesocosms (76 × 46 × 14 cm; 20‐L oval tanks; called hereafter “streams”) housed in a greenhouse. Natural water from a deep, mesotrophic reservoir (Horsetooth Reservoir, Larimer County, CO, USA) was delivered to each stream at a rate of 1.0 L/min. The water quality in control streams was typical of Colorado high mountain streams; low hardness (~30 mg/L CaCO_3_) and dissolved organic carbon (2.4 mg/L; not measured in the present study, but see Naddy et al., 2007), and circumneutral pH. A constant current velocity of 0.35 m/s was maintained by paddlewheels in each stream mesocosm. Colonized trays were randomly assigned to 1 of 18 stream mesocosms (three trays/a stream mesocosm). After a 24‐h acclimation period, we started 10‐day exposure experiments using calibrated peristaltic pumps that delivered stock solutions from 20‐L carboys (10 ml/min). Previous experiments conducted in the same facility have shown that a 10‐day exposure to metals was sufficient to demonstrate significant toxicity (Clements et al., 2019).

In the Cu + Ca mesocosm experiment (late September 2012), macroinvertebrate communities were exposed in triplicate (3 streams/treatment level) to a target concentration of Cu (25 μg/L) across a gradient of Ca concentrations (∼10–100 mg/L, resulting in water hardness ranging from 30 to 250 mg/L CaCO_3_; Table 1). Stock solutions were prepared using analytical grade CuSO_4_·5H_2_O, and/or CaCl_2_ dissolved in the reservoir water. After the 10‐day exposure, macroinvertebrates retained by a 350‐μm sieve were preserved in 80% ethanol for enumeration at a later date. Organisms were identified to the lowest practical level of taxonomic resolution (typically genus or species for most organisms; subfamily for chironomids). We did not count the number of adult insects that emerged from the experimental streams; however, adult emergence was very low during these experiments and would not have affected our findings. To ensure that Ca alone had no adverse effects on aquatic insects, a second “Ca‐only” mesocosm experiment was conducted in October 2012 with two treatments: control versus 100 mg/L Ca  (Table 1). Macroinvertebrate trays were collected after 32 days of colonization as just described and transferred to the experimental stream facility on October 6, 2012. All other experimental details were the same as in the first experiment.

**Table 1:** Water quality characteristics (mean ± standard error) in two stream mesocosm experiments

Treatment	Cu (μg/L)	WQC_hardness_ (μg/L)	WQC_BLM_ (μg/L)	Ca (mg/L)	Water hardness (mg/L CaCO_3_)	Conductivity (μS/cm)
Cu and Ca experiment						
Control	3.2 ± 0.6	3.1	8.7	9 ± 0.04	29 ± 0.1	64 ± 0.2
Cu only	24.7 ± 0.4	3.1	8.7	9 ± 0.1	29 ± 0.2	64 ± 0.3
Cu + hardness 50 mg/L	26.0 ± 1.1	5.0	7.9	18 ± 0.5	50 ± 1.2	116 ± 4
Cu + hardness 100 mg/L	29.1 ± 0.6	9.3	6.9	41 ± 1	108 ± 2.0	240 ± 6
Cu + hardness 150 mg/L	26.7 ± 1.0	13	6.8	59 ± 3	154 ± 8.0	361 ± 21
Cu + hardness 250 mg/L	26.7 ± 1.4	20	7.2	99 ± 2	253 ± 5.5	564 ± 9
Ca‐only experiment						
Control	3.5 ± 1.2	3.4	7.0	10 ± 1	32 ± 3.1	58 ± 0.2
Hardness 250 mg/L	4.9 ± 0.7	20	7.3	102 ± 5	261 ± 13.7	511 ± 24

WQC_hardness_, hardness‐adjusted Environmental Protection Agency (USEPA, 2002) environmental water quality criteria for copper; WQC_BLM_, biotic ligand model‐based USEPA (2002, 2007) environmental water quality criteria for copper calculated using BLM Freshwater and Marine Ver 3.41.2.45 (Windward Environmental, 2022); see the Supporting Information, Table S2 for the water quality parameters used).

### Chemical analysis

Water temperature, pH, dissolved oxygen, and conductivity were measured on multiple occasions using hand‐held meters (models 550A and 63; YSI; see the Supporting Information, Table S1). Water samples (0.5 L) were collected three times (i.e., Days 4, 6 [or 7], and 9) to measure water hardness and alkalinity in the laboratory using standard titration procedures (American Public Health Association, American Water Works Association, & Water Pollution Control Federation, 1980). Water samples (15 ml) for analysis of dissolved metals (Cu, Ca, and magnesium [Mg]) were collected three to four times during the experiments and filtered through a 0.45‐μm filter and acidified to a pH of less than 2.0 with ultrapure nitric acid. Metal concentrations were analyzed using inductively coupled plasma–optical emission spectrometry with a PerkinElmer Optima 5300 DV. For a more detailed quality assurance/quality control schedule, see Cadmus et al. (2016). Because the CaCO_3_‐equivalent water hardness values measured by the titration and calculated by the measured concentrations of Ca and Mg varied little, the latter values were hereafter used in the present study.

### Data analysis

Macroinvertebrate community metrics including total taxon richness (the total number of taxa), mayfly (Ephemeroptera) richness, total abundance (the total number of individuals), mayfly abundance, stonefly (Plecoptera) abundance, caddisfly (Trichoptera) abundance, and abundances of nine dominant taxa were calculated to examine the responses in the two mesocosm experiments. The nine dominant taxa in the two mesocosm experiments were *Baetis* sp. (Baetidae, Ephemeroptera), *Epeorus* sp. (Heptageniidae, Ephemeroptera), *Utacapnia* sp. (Capniidae, Plecoptera), *Amphinemura* sp. (Nemouridae, Plecoptera), *welts asp*. (Chloroperlidae, Plecoptera), *Isoperla* sp. (Perlodidae, Plecoptera), *Lepidostoma* sp. (Lepidostomatidae, Trichoptera), Chironomidae (Diptera), and *Heterlimnius corpulentus* (Elmidae, Coleoptera). Abundances were log_10_(*x* + 1) transformed to satisfy the assumptions of the statistical analysis described in the next paragraph.

Although the two mesocosm experiments were separated by more than 30 days, control‐stream community composition was slightly different between experiments, and therefore the results were analyzed separately. For the Cu and Ca experiment, a multiple linear regression analysis was used to examine the effects of increasing concentrations of Cu and Ca on the macroinvertebrate metrics. We used multiple linear regression analysis rather than two‐way analysis of variance because we expected the protective effects of increasing Ca concentrations to increase linearly, as observed in laboratory toxicity tests (Crémazy et al., 2017; De Schamphelaere & Janssen, 2002). In these models, measured (dissolved) concentrations of Cu and Ca were included as the predictors. For the Ca‐only experiment, the differences between two treatments were examined by the Student's *t*‐test. All the statistical analyses were performed using R Ver 3.5.1 (R Core Team, 2018), and a significance level (*α*) of 0.05 was used. All the macroinvertebrate data are available in the Supporting Information.

## RESULTS

### Water quality characteristics

Mean dissolved concentrations of Cu in Cu‐added treatments were 25–29 μg/L, which were close to or slightly higher than the target concentration of 25 μg/L (Table 1). Mean values of water hardness and Ca concentration in streams without Ca addition were approximately 30 and 10 mg/L, respectively. Measured concentrations of water hardness and Ca in the Ca‐added streams increased to approximately 250 and 100 mg/L, respectively. Other water quality characteristics (alkalinity, Mg, dissolved oxygen) measured in stream mesocosms were similar between experiments, but water temperature was approximately 3 °C cooler in the second Ca‐only experiment conducted approximately 1 month later in the fall. As expected, pH was neutral in all the streams (range: 7.6–7.9) but tended to be slightly lower in the Ca‐added streams (Supporting Information, Table S1). The corresponding hardness‐adjusted WQC values for Cu increased from 3.1 up to 20 μg/L with increased water hardness, although the BLM‐based WQC values varied little (6.8–8.7 μg/L; Table 1). The dissolved concentrations of Cu in control streams were close to the hardness‐adjusted WQC values (~3 μg/L), but were well below the BLM‐based WQC values (more than 7 μg/L). The explanation for these slightly elevated concentrations of Cu in control streams compared with previous experiments using water from this same reservoir (Naddy et al., 2015) is unknown.

### Responses of benthic communities to Cu and Ca

**Figure 1:**
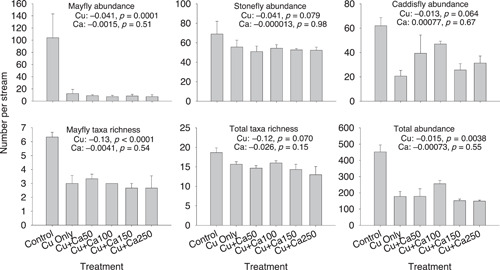
Mean taxon richness and abundances of macroinvertebrate communities in individual treatments of the copper (Cu) and calcium (Ca) experiment (see Table 1 for details of the treatments). Error bars are +1 × standard errors. Estimated regression coefficients and corresponding *p* values for Cu and Ca concentrations are shown in each panel.

**Figure 2:**
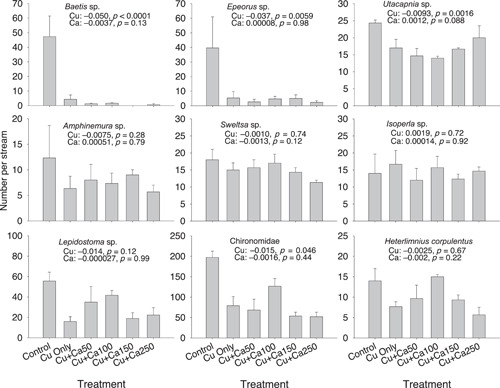
Mean abundances of nine macroinvertebrate dominant taxa in individual treatments of the copper (Cu) and calcium (Ca) experiment (see Table 1 for details of the treatments). Error bars are +1 × standard errors. Estimated regression coefficients and corresponding *p* values for Cu and Ca concentrations are shown in each panel.

The effects of Cu on mayfly richness, total abundance, and mayfly abundance were highly significant, reducing these metrics in the Cu‐only treatments by 54%, 61%, and 91%, respectively (Figure 1). Total taxa richness and total abundance of stoneflies and caddisflies were reduced by 21%–51% and approached statistical significance in the Cu‐only treatments. In contrast to these results, the addition of Ca had no significant effects on any of these six community metrics. Similarly, although abundances of four of the dominant taxa (i.e., *Baetis*, *Epeorus*, *Utacapnia*, and Chironomidae) were significantly reduced by Cu (mean reduction: 32%–96%), we did not observe a protective effect of Ca on any taxa (Figure 2).

In the Ca‐only mesocosm experiment there were no significant differences in any of the six community metrics between controls and Ca‐added streams (Table 2). Indeed, values of all 15 macroinvertebrate metrics we examined were similar between the two treatments, with the ratios of metric values in Ca‐added to control streams ranging between 0.8 and 1.6.

**Table 2:** Macroinvertebrate metrics on richness and abundance (mean ± standard error) in the Ca‐only experiment

	Treatment			
Invertebrate metric	Control	Ca added	*t*	*p* value
Total taxon richness	22 ± 2	21 ± 1	0.28	0.79
Mayfly richness	7 ± 0.3	8 ± 0.3	−0.71	0.52
Total abundance	564 ± 50	539 ± 42	0.39	0.72
Mayfly abundance	283 ± 25	270 ± 9	0.50	0.64
Stonefly abundance	57 ± 7	69 ± 1	−1.69	0.17
Caddisfly abundance	53 ± 8	44 ± 7	0.91	0.41
Dominant taxa				
*Baetis* sp.	53 ± 8	48 ± 3	0.57	0.60
*Epeorus* sp.	202 ± 15	193 ± 11	0.45	0.68
*Utacapnia* sp.	11 ± 1	13 ± 1	−1.60	0.18
*Amphinemura* sp.	7 ± 3	8 ± 1	−0.30	0.78
*Sweltsa* sp.	16 ± 3	25 ± 3	−2.35	0.08
*Isoperla* sp.	20 ± 4	17 ± 4	0.51	0.64
*Lepidostoma* sp.	46 ± 6	37 ± 6	1.02	0.36
Chironomidae	155 ± 18	141 ± 43	0.30	0.78
*Heterlimnius corpulentus*	10 ± 2	11 ± 1	−0.45	0.67

Values of *t* and *p* were obtained from two‐sample *t* tests.

## DISCUSSION

Exposure of aquatic insect communities to 25 μg/L Cu for 10 days in stream mesocosm experiments resulted in significant decreases in total abundance, number of taxa, and abundance of many macroinvertebrate taxa. As demonstrated in previous experiments (Clements et al., 2013), mayflies (Ephemeroptera) were especially sensitive to Cu exposure. Total abundance of mayflies and abundance of several highly sensitive taxa (e.g., *Baetis* and *Epeorus*) were reduced by 88%–96% in Cu‐only treatments compared with controls. Although dissolved concentrations of Cu in Cu‐added streams were higher than the hardness‐adjusted WQC for Ca‐added streams (Table 1), we predicted that effects of Cu on macroinvertebrates metrics should have been reduced by the addition of Ca.

In contrast to our expectations, any significant influence of Ca was not detected for any of the metrics we examined (Figures 1 and 2). These results indicated that the addition of Ca and the associated increase in water hardness up to 250 mg/L CaCO_3_ did not mitigate effects of Cu on macroinvertebrate communities. Although protective effects of Ca against Cu toxicity have been often observed in acute and chronic toxicity tests (Crémazy et al., 2017; De Schamphelaere & Janssen, 2002; Ha et al., 2017; Howarth & Sprague, 1978; Santore et al., 2001; USEPA, 2007), other studies have shown that increasing Ca or hardness concentrations provided little or no protective effect from Cu toxicity (De Schamphelaere et al., 2003; De Schamphelaere & Janssen, 2004; Markich et al., 2005, 2006; Villavicencio et al., 2011; Wang et al., 2009; Winner, 1985). Interestingly, an 18‐month lotic mesocosm experiment conducted in a hard water (342 mg/L CaCO_3_) demonstrated that a Cu concentration of 20 μg/L led to statistically significant effects on the zooplankton and macroinvertebrate communities (Joachim et al., 2017), which is consistent with our findings. Because empirical (particularly experimental) evidence demonstrating the protective effects of Ca and other water quality variables on macroinvertebrate communities is very limited (but see Kashian et al., 2004), additional experiments are required to understand the influence of modifying factors on the toxicity and bioavailability of metals to these organisms.

The lack of a protective effect of Ca on Cu toxicity observed in our mesocosm experiments appears to be inconsistent with the increased hardness‐adjusted WQC for Cu (Table 1). However, the USEPA (2007) suggests that the hardness‐adjusted WQC can be underprotective if water hardness, alkalinity, and pH do not covary, which was the situation in our experiments. In natural systems, a positive relationship among water hardness, alkalinity, and pH is generally observed in rivers worldwide (Hartmann et al., 2019; see the Supporting Information, Figure S1). Despite an approximately 10× increase in water hardness across Ca treatments in our study, BLM‐based WQC values showed little variation among treatments and actually decreased at higher water hardness, likely because of the marginally lower pH values (Table 1). Although our study was not designed to estimate the protectiveness of the BLM‐based WQC for Cu, the model underlying the BLM‐based WQC is consistent with our results because it accurately predicted that increased Ca concentrations would have little protective effects. Our findings suggest that the calculation of both hardness‐adjusted and BLM‐based WQC would be valuable in systems in which changes in water hardness of natural waters do not covary with changes in pH and alkalinity (USEPA, 2007).

The effects of dissolved ions and the associated increases in conductivity and ionic strength on aquatic insects have been measured in field studies (Cormier et al., 2013) and in mesocosm experiments (Clements & Kotalik, 2016). A field‐derived benchmark of 300 µS/cm specific conductance has been proposed to protect the most sensitive aquatic insects in naturally low conductivity streams. Although specific conductance in our highest Ca treatments exceeded this benchmark, we did not see evidence of negative effects on benthic communities. The second mesocosm experiment, in which organisms were exposed to 261 mg/L Ca (511 μS/cm), had no effects on macroinvertebrate metrics or abundance of any of the dominant taxa in our study. These results demonstrate that the similarity of the Cu‐only and the Cu + Ca treatments in the first mesocosm experiment did not result from the negative effects of greater ionic strength associated with Ca additions.

Several researchers have noted the discrepancies between responses of aquatic insects to metals in short‐term laboratory toxicity tests compared with those measured in the field (Brix et al., 2011; Cadmus et al., 2020; Clements et al., 2013; Iwasaki et al., 2018; Mebane et al., 2020b). The data available on effects of metals derived from short‐term laboratory experiments suggest that aquatic insects are highly tolerant to metals. In contrast, field studies of streams receiving metals have shown that some aquatic insects are often among the first group eliminated. Likely explanations for these differences include the short duration of laboratory toxicity tests, the difficulties in conducting laboratory tests with organisms known to be highly sensitive to metals (e.g., many Ephemeroptera), and the failure to account for other routes of metal exposure (e.g., diet; Cadmus et al., 2020; Clements et al., 2013; Iwasaki et al., 2018; Mebane et al., 2020b). A better understanding of the physiological, morphological, and behavioral factors that determine the sensitivity of some aquatic insects to metals and the water quality characteristics that influence metal bioavailability to these organisms is essential for predicting effects in the field.

## Supporting Information

The Supporting Information is available on the Wiley Online Library at https://doi.org/10.1002/etc.5308.

## Author Contributions Statement

**Yuichi Iwasaki:** Conceptualization; Data curation; Formal analysis; Funding acquisition; Investigation; Methodology; Visualization; Writing—original draft, review, and editing. **Pete Cadmus:** Conceptualization; Data curation; Formal analysis; Investigation; Methodology; Writing—review and editing. **James Ranville:** Funding acquisition; Investigation; Methodology; Resources; Writing—review and editing. **William H. Clements:** Conceptualization; Data curation; Funding acquisition; Investigation; Methodology; Supervision; Visualization; Writing—review and editing.

## Supporting information

This article includes online‐only Supporting Information.

Supporting information.

Supporting information.

## Data Availability

Data, associated metadata, and calculation tools are available from the corresponding author (yuichiwsk@gmail.com).
