# Supplementary material for: Stream Mesocosm Experiments Show no Protective Effects of Calcium on Copper Toxicity to Macroinvertebrates
Source: Environ Toxicol Chem. 2022 Mar 21;41(5):1304–10. doi: 10.1002/etc.5308 (PMC9311704; doi:10.1002/etc.5308)
Supplement: Supplementary file 1 — Supporting information. [file ETC-41-1304-s001.docx]

**Supporting Information for “Stream mesocosm experiments show no protective effects of calcium on copper toxicity to macroinvertebrates”**

Table S1. Other water quality characteristics (mean ± standard error) in two stream mesocosm experiments.

| Treatment | Temperature | pH | DO | Alkalinity | Mg |
| --- | --- | --- | --- | --- | --- |
|  | °C |  | mg/L | CaCO_3_ mg/L | mg/L |
| Cu & Ca experiment |  |  |  |  |  |
| Control | 17.6 ± 0.02 | 7.90 ± 0.11 | 7.72 ± 0.01 | 30.1 ± 0.1 | 1.47 ± 0.01 |
| Cu only | 17.6 ± 0.05 | 7.90 ± 0.02 | 7.38 ± 0.02 | 29.8 ± 0.2 | 1.49 ± 0.01 |
| Cu + Hardness 50 mg/L | 17.6 ± 0.02 | 7.82 ± 0.05 | 7.39 ± 0.01 | 29.8 ± 0.1 | 1.49 ± 0.02 |
| Cu + Hardness 100 mg/L | 17.6 ± 0.03 | 7.65 ± 0.05 | 7.36 ± 0.04 | 29.8 ± 0.3 | 1.50 ± 0.02 |
| Cu + Hardness 150 mg/L | 17.6 ± 0.03 | 7.60 ± 0.03 | 7.40 ± 0.02 | 30.3 ± 0.1 | 1.49 ± 0.01 |
| Cu + Hardness 250 mg/L | 17.5 ± 0.01 | 7.56 ± 0.03 | 7.42 ± 0.02 | 30.4 ± 0.3 | 1.47 ± 0.02 |
|  |  |  |  |  |  |
| Ca only experiment |  |  |  |  |  |
| Control | 14.9 ± 0.14 | 7.73 ± 0.06 | 8.16 ± 0.02 | 29.8 ± 0.1 | 1.51 ± 0.07 |
| Hardness 250 mg/L | 14.7 ± 0.02 | 7.59 ± 0.04 | 8.19 ± 0.01 | 29.6 ± 0.1 | 1.54 ± 0.04 |

Water temperature, pH, and dissolved oxygen (DO) as well as conductivity (see Table 1) were measured on days 3, 4, 5, 7, 8, and 9 in the first experiment and on days 4, 6, and 9 in the second experiment.

Table S2. Water quality parameters used for calculating BLM-based water quality criteria.

| Treatment | Temperature | pH | DOC | Ca | Mg | Na | K | SO4 | Cl | Alkalinity |
| --- | --- | --- | --- | --- | --- | --- | --- | --- | --- | --- |
|  | °C |  | mg/L | mg/L | mg/L | mg/L | mg/L | mg/L | mg/L | mg/L |
| Cu & Ca experiment |  |  |  |  |  |  |  |  |  |  |
| Control | 17.6 | 7.90 | 2.4 | 9.08 | 1.47 | 3.5 | 0.5 | 5.6 | 1.9 | 30.1 |
| Cu only | 17.6 | 7.90 | 2.4 | 9.18 | 1.49 | 3.5 | 0.5 | 5.6 | 1.9 | 29.8 |
| Cu + Hardness 50 mg/L | 17.6 | 7.82 | 2.4 | 17.6 | 1.49 | 3.5 | 0.5 | 5.6 | 16.8 | 29.8 |
| Cu + Hardness 100 mg/L | 17.6 | 7.65 | 2.4 | 40.8 | 1.5 | 3.5 | 0.5 | 5.6 | 57.9 | 29.8 |
| Cu + Hardness 150 mg/L | 17.6 | 7.60 | 2.4 | 59.3 | 1.49 | 3.5 | 0.5 | 5.6 | 90.6 | 30.3 |
| Cu + Hardness 250 mg/L | 17.5 | 7.56 | 2.4 | 98.7 | 1.47 | 3.5 | 0.5 | 5.6 | 160.3 | 30.4 |
| Ca only experiment |  |  |  |  |  |  |  |  |  |  |
| Control | 14.9 | 7.73 | 2.4 | 10.3 | 1.51 | 3.5 | 0.5 | 5.6 | 1.9 | 29.8 |
| Hardness 250 mg/L | 14.7 | 7.59 | 2.4 | 102 | 1.54 | 3.5 | 0.5 | 5.6 | 164.0 | 29.6 |

Values of DOC (dissolved organic concentration), concentrations of Na, K, SO_4_ and Cl were obtained from [Naddy et al. (2007)](#_ENREF_30). Increased concentrations of chloride ion were calculated based on the increased Ca concentrations by assuming that the added CaCl_2_ were completely dissolved. For the BLM calculation, the following default log K values for biotic ligand (BL) were used: log K_BL-Cu_ = 7.4, log K_BL-CuOH_ = –1.3, log K_BL-Ca_ = 3.6, log K_BL-Mg_ = 3.6, log K_BL-H_ = 5.4, log K_BL-Na_ = 3.0.

Figure S1. Relationships between water hardness, and alkalinity and pH.

These scatter plots were generated based on the data available in Hartmann et al. (2019). For the analysis, negative values of alkalinity (μeq/L) were removed from the original data, water hardness was calculated based on Ca and Mg concentrations, and site means were calculated for these water quality parameters. When calculating Pearson’s correlations, water hardness and alkalinity were log_10_-transformed.
